# Using Participatory Mapping to Diagnose Upstream Determinants of Health and Prescribe Downstream Policy-Based Interventions

**DOI:** 10.5888/pcd17.200123

**Published:** 2020-11-05

**Authors:** Jason A. Douglas, Andrew M. Subica, Laresha Franks, Gilbert Johnson, Carlos Leon, Sandra Villanueva, Cheryl T. Grills

**Affiliations:** 1 Chapman University, Crean College of Health and Behavioral Sciences, Department of Health Sciences, Orange, California; 2 University of California, Riverside School of Medicine, Department of Social Medicine, Population, and Public Health, Riverside, California; 3 Community Coalition, Los Angeles, California; 4 Loyola Marymount University, Psychology Applied Research Center, Los Angeles, California

## Abstract

Participatory mapping is a powerful methodology for working with community residents to examine social and environmental determinants of public health disparities. However, this empowering methodology has only been applied sparingly in public health research and practice, with limited examples in the literature. To address this literature gap, we 1) review participatory mapping approaches that may be applied to exploring place-based factors that affect community health, and 2) present a mixed-methods participatory geographic information systems (PGIS) examination of neighborhood assets (eg, streetlights) and challenges (eg, spaces of crime and violence) related to access to public parks in South Los Angeles, California. By taking a participatory, fine-grained spatial approach to examining public park access with input from 40 South Los Angeles adolescent and adult residents, our community-engaged PGIS approach identified tobacco shops as previously unrecognized community institutions that are associated with increased neighborhood crime and violence. Our investigation revealed unique challenges in community-level public park access that would likely have been overlooked by conventional spatial epidemiology and social science methods, such as surveys and questionnaires. Furthermore, our granular community-informed approach supported resident and stakeholder advocacy efforts toward reducing the proliferation of tobacco shops through community organizing and policy change initiatives. We thus contend that it would benefit public health research and practice to further integrate empowering, grassroots-based participatory mapping approaches toward informing advocacy efforts and policies that promote health and well-being in disadvantaged communities.

**Summary**

**What is already known on this topic?**

Systemic social and environmental inequities contribute to uneven health outcomes in disadvantaged communities. Participatory mapping is a powerful visual methodology for partnering with affected communities to develop grounded understandings of community health assets and challenges.

**What is added by this report?**

We demonstrate use of participatory mapping as a potent methodology for diagnosing place-based determinants of health disparities, review participatory mapping approaches for public health research and practice, and present a case example of this methodology.

**What are the implications for public health practice?**

Participatory mapping is ideally suited to diagnosing upstream determinants of health and prescribing downstream policy interventions that attend to resident interests and needs.

## Introduction

Population health is fundamentally connected to the conditions in which people live, work, learn, and play (1). This phenomenon is apparent in disadvantaged communities that experience adverse work and neighborhood conditions and unequal access to health-promoting resources (2,3). For example, in the United States, inequities in public park access exist between disadvantaged communities and more privileged communities (4). Neighborhood walkability in disadvantaged communities is also spatially uneven because of numerous neighborhood-level challenges, including damaged sidewalks, physical disorder (eg, litter, vandalism), and crime (5,6). Investigations have advanced an understanding that place-based inequities (eg, public park and neighborhood walkability inequities) frequently contribute to health disparities (eg, obesity, diabetes) in disadvantaged communities (5,7).

Participatory visual methodologies born of community–academic partnerships have been instrumental in identifying these and other place-based inequities in disadvantaged communities (8). Visual methodologies — eg, photovoice, participatory mapping — encourage collaborative, democratic modes of knowledge production, challenging the notion that research is only for highly trained experts (9,10). Community organizers have used visual methodologies for decades to gauge resident concerns and needs and inform systems and policy change. Participatory mapping is a particularly potent, visual methodology for public health, as it provides a rigorous, place-based, community-engaged approach for investigating the root causes of health disparities and a framework for expanding investigations via complementary methodologies (eg, geographic information systems [GIS]) (11–13). Participatory mapping responds to the marginalized voices and interests of community residents by involving them in collaborative map production, from drawing maps on the ground to using GIS-based map creation approaches, toward investigating community concerns and needs in general (eg, walkability) and specific (eg, neighborhood) built environments (11,14,15). Thus, participatory mapping is well designed for eliciting spatial *and* social knowledge grounded in residents’ experience of place (16). Participatory mapping consequently provides the ideal methodologic approach to develop grounded understandings of community health assets and challenges in disadvantaged communities (13,17). For example, Freund et al (11) partnered with LGBTQ young adults to map health-seeking behaviors — identifying churches, police stations, and bars as spaces young adults relied on to garner information about health services. In another study, community residents identified local beaches as spaces of HIV risk in Kenya (12). A third example engaged community residents in the collection and analysis of “fine-grained, street-level, actionable data” about health challenges associated with illegal dumping and faulty storm water infrastructure in northwest Atlanta, Georgia (13). These findings illustrate the potential of participatory mapping for identifying and elucidating the mechanisms underlying patterns observed through large-scale spatial epidemiology. Yet, despite these powerful examples, participatory mapping has only been applied sparingly in public health, with nominal case examples and literature to inform research and practice.

Given the promising public health applicability of participatory mapping, we address a glaring literature gap by 1) providing an overview of participatory mapping approaches for public health and 2) illustrating the potential of participatory mapping by presenting a case example of community health asset and challenge mapping conducted as part of a Centers for Disease Control and Prevention (CDC) Racial and Ethnic Approaches to Community Health (REACH) project that was instrumental in diagnosing upstream determinants of health (eg, crime, violence) and prescribing downstream policy-based interventions.

## Participatory Mapping Approaches for Public Health

Participatory mapping mirrors community-based participatory research and citizen science (18), with the understanding that place-based research benefits from including community members (9). Thus, because place-based inequities often manifest in health disparities (5,7), participatory mapping in the public health context is ideally suited for identifying actionable data to inform systems and policy changes that are attentive to health and place (13).

**Hands-on mapping. **An effective approach for working in low-resource settings is hands-on mapping, which has been applied to a range of health issues such as mapping arsenic exposure (19), access to health care services (11), and broader community health assets and challenges (20). In the hands-on approach, participants draw maps on the ground or paper, for example, about their immediate spatial health concerns. These maps are useful for gathering exploratory data to frame public health issues based on community interests. Furthermore, as a visual methodology, hands-on mapping is useful for engaging communities across languages and literacy rates (19).

**Participatory mapping with reference maps.** Participatory mapping with reference maps engages residents with scaled maps that reflect the relationship between a unit of measurement on a map and related distance on the ground (eg, street maps) to represent and explicate features of the social and built environment that affect public health. Methodologically, reference maps are particularly well suited to eliciting fine-grained spatial data, because they provide accurate geographical depictions of research sites that can easily be transferred to geospatial analysis platforms (eg, ArcMap, Geoda) to analyze community-identified interests from spatial epidemiology perspectives. This approach has been particularly effective in identifying community access to health services (16), spaces of crime and violence (14), and exposure to air pollution (21).

**Participatory geographic information systems (PGIS). **With an emphasis on coproduction of knowledge found in community-based participatory research (22), participatory geographic information systems (PGIS) provides a mixed-methods approach (23) and framework for residents and trained spatial researchers to collaboratively integrate local knowledge with technical geospatial approaches (15). Geospatial research can require extensive training in complex methodologies and software systems (eg, ArcMap, Q); PGIS is a powerful alternative that combines grounded resident knowledge with GIS practitioner expertise. For example, PGIS may be used to combine primary data (eg, hands-on mapping of pollution exposure) and secondary data (eg, asthma-related emergency department visits) to accurately diagnose fundamental causes of disease. This mode of stakeholder engagement is well suited to investigating a range of spatially endemic public health challenges, such as food insecurity (24), access to health care (25), and exposure to noise pollution (21).

## Racial and Ethnic Approaches to Community Health — Participatory Mapping in Context

To illustrate the public health research potential of participatory mapping, we present an exploratory sequential PGIS case example that combined participatory mapping with reference maps and GIS from our multifaceted community-based participatory research initiative in South Los Angeles, California. This community is predominantly Latinx (64%) and African American (31%) with inequitable access to public parks, a feature commonly associated with community health (26). Led by South Los Angeles residents via the Community Coalition, a community-based nonprofit organization, our CDC-funded initiative aimed to reduce obesity and associated adverse health outcomes by improving resident access to 1) health care exchanges, 2) community health clinics, and 3) physical activity resources (eg, public parks). Guided by the available literature and resident feedback, we developed participatory mapping procedures (Table 1) to examine the accessibility of physical activity resources in South Los Angeles.

**Table 1:** Description of Participatory Mapping Steps, South Los Angeles, 2015

Steps	Description
1. Establish community partnerships	Participatory mapping and broader community-based participatory research initiatives are ideally supported by community-level partnerships between organizations that 1) have the capacity to generate resident participation, 2) have the requisite insight to point out the nuanced layers of community context and issues, and 3) are well positioned to collaboratively implement public health advocacy and promotion initiatives (27).
2. Establish a community-driven research agenda and identify geographic scope	Because community residents are central to the participatory mapping process, the research agenda should be driven by resident interests and needs specific to an identifiable geographic area (22).
3. Recruit mapping participants	Participatory mapping is effectively driven by resident knowledge of place. Therefore, it will benefit communities of practice using this methodology to develop a recruitment strategy that 1) is informed by the available literature, and 2) engages participants possessing a grounded knowledge of the research site (12).
4. Conduct participatory mapping sessions	In this central phase of the participatory mapping process, facilitators should engage participants with mapping tools that are ideally suited to organizational capacity and familiar to participants, such as paper maps and online mapping resources.
5. Digitize maps and document resident interests	Digitizing maps to create an overview of resident interests and concerns, and documenting resident identified place-based determinants of health will be beneficial for participant review and informing ensuing analyses.
6. Reconvene residents for map review	Residents review data and provide additional data points when deemed necessary.
7. Scale maps to larger geographic area	Participatory mapping is typically conducted at a small scale (eg, within a 1-mile buffer). Therefore, to examine convergent validity, communities of practice may further examine relationships between variables (eg, tobacco shops and crime) on a larger scale via GIS.

### Steps in the PGIS case example

#### Step 1: Establish community partnerships

The Community Coalition, which has been organizing residents in South Los Angeles for health equity since 1990, established strategic partnerships to build research capacity and develop data-driven health promotion interventions. The Psychology Applied Research Center (PARC) at Loyola Marymount University provided research capacity, while St. John’s Well Child and Family Centers and To Help Everyone Health and Wellness Centers provided health care services. Partnerships with Advocates for Peace and Urban Unity, Reclaiming America’s Communities through Empowerment, and 3 Wins Fitness served to improve public park safety and recreational programming. Finally, the Los Angeles County Department of Public Health–Injury and Violence Prevention Program assisted with data acquisition.

#### Step 2: Establish a community-driven research agenda and identify geographic scope

**Figure 1:**
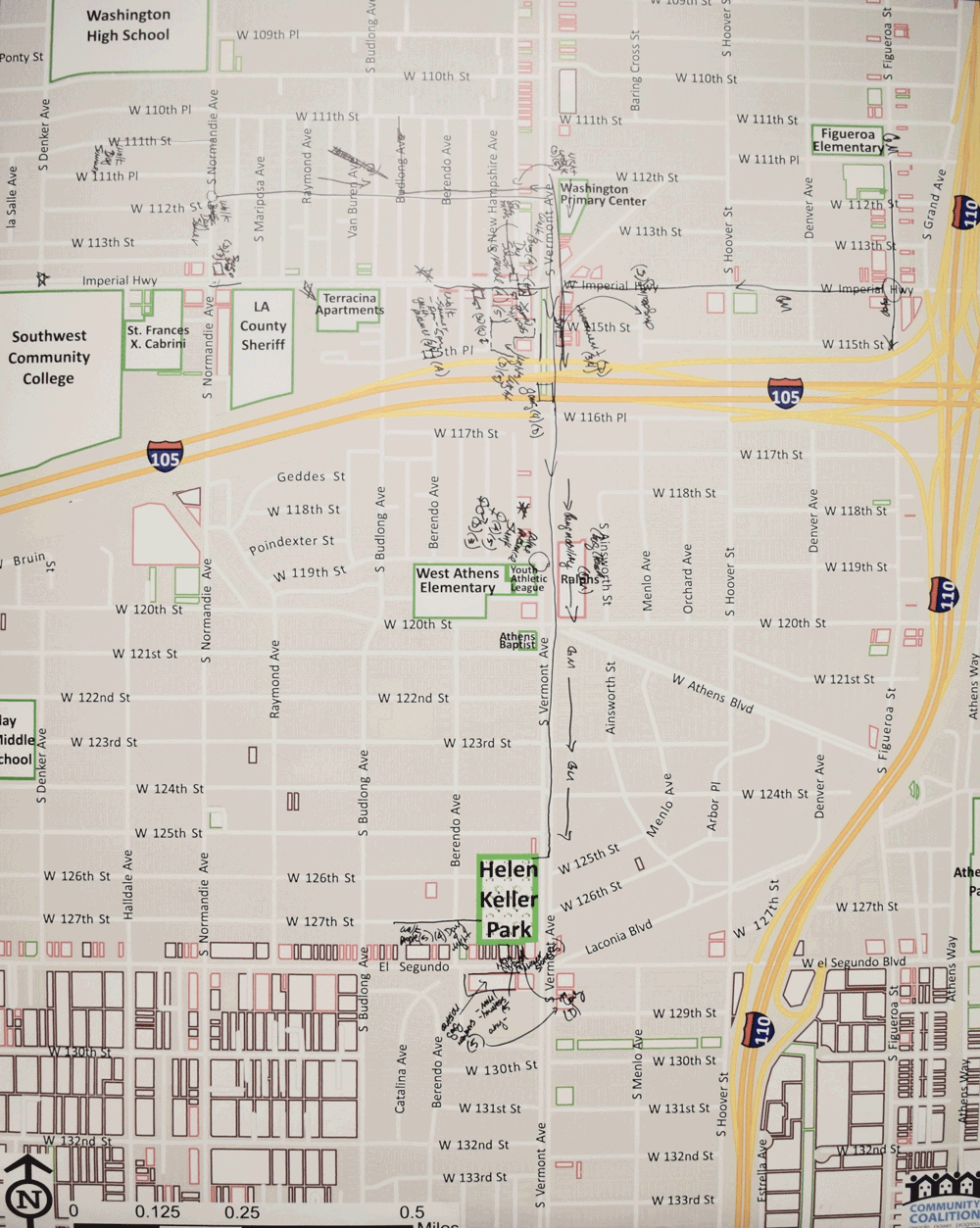
Paper street map of community park access assets and challenges, South Los Angeles, 2015.

Public parks in South Los Angeles are often underused because of resident concern about nearby crime and safety issues (28). Therefore, to examine access to physical activity resources, our team leveraged participatory mapping with reference maps to 1) qualitatively investigate place-based factors affecting resident access to public parks, 2) provide a framework for applying quantitative GIS analyses, and 3) support evidence-informed interventions and residents’ advocacy efforts. We also took a recreational justice approach (29) to develop an understanding of place-based determinants of physical activity by examining resident access to Helen Keller Park in the Westmont/West Athens area of South Los Angeles. The team developed 4-by-5-foot paper street maps in ArcGIS 10.3 to map resident mobility routes to the park. Our maps detailed well-known neighborhood institutions such as schools, law enforcement centers, community clinics, and Helen Keller Park (Figure 1).

#### Step 3: Recruit mapping participants

Public park use tends to decline in high-crime areas (30). We selected the following participant inclusion criteria to reflect the diversity of South Los Angeles: 1) adolescent and adult residents who spoke English and/or Spanish, and 2) lived within a half-mile buffer of Helen Keller Park, a high-crime area. The Community Coalition used a form of nonrandomized cluster sampling to recruit 40 adolescent and adult residents from their community base of predominantly African American/Black and Latinx supporters who possessed a knowledge of community context. Random sampling of mapping participants was deemed inappropriate because of the necessity of recruiting residents who were familiar with Helen Keller Park and had a vested interest in improving access to the park and community safety (31).

#### Step 4: Conduct participatory mapping sessions

Trained Community Coalition and PARC mapping facilitators and South Los Angeles residents convened at a local community center in March 2015 to map 1) resident walking routes to Helen Keller Park and 2) neighborhood characteristics (eg, streetlights, crime) associated with park access. This information would then be scaled up to 3) examine place-based determinants of health across South Los Angeles, and based on these results, 4) develop grassroots solutions for improving recreational access.

To achieve mapping objectives, as a collective group, residents were briefed on mapping procedures and informed that they could stop participating at any time. Participants then joined 1 of 4 contemporaneous, 1-hour mapping sessions, each with 8 to 12 participants. Two mapping sessions were organized according to resident location to account for geographic differences in resident experiences, a third session accommodated Spanish-language speakers, and a fourth session included community adolescents aged 15 to 18. In addition, public health officials and gang interventionists, who also possessed knowledge of the area, aided in session facilitation.

Facilitators began each session by reviewing mapping objectives. Participants were then geographically oriented to recognizable neighborhood institutions on the 4-by-5-foot paper street maps (Figure 1) to confirm participant map awareness. Following this step, participants were asked to identify their walking and public transportation routes to Helen Keller Park and neighborhood assets (eg, streetlights) and challenges (eg, crime) that may encourage or encumber resident mobility. Mapping facilitators traced resident mobility routes, marking assets and challenges along each route. Notes were also recorded on paper to capture resident concerns that could not be mapped. Then, participants reconvened into the collective group to review overarching themes and provide an opportunity for participants to add any additional points missed during breakout sessions. Participants were offered dinner after the event.

#### Step 5: Digitize maps and document resident interests

The mapping sessions revealed 5 assets and 14 challenges (Table 2). Among the challenges, residents emphasized the presence of “nuisance properties” (ie, properties with a negative effect on public health and safety) associated with crime and violence as a priority concern curtailing park access and community-wide safety. These nuisance properties included 1) off-sale alcohol outlets, 2) tobacco shops, 3) medical marijuana dispensaries (MMDs), and 4) “problem” motels.

**Table 2:** Community Assets and Challenges Identified by Residents During Participatory Mapping Sessions, South Los Angeles, 2015

Type	Description
**Assets**	
Community presence	Areas where residents have clear view of the surrounding environment
Street lighting	Well-lighted areas with surrounding environments visible at night
Los Angeles sheriff	Los Angeles Sheriff Station and patrol routes
Security cameras	Mounted security cameras with clear view of the surrounding environment
Bus stations	Areas where residents often congregate
**Challenges**	
Drug use	Areas synonymous with public drug use
Drug dealing	Areas synonymous with public drug dealing
Vacant lots	Abandoned, often poorly lighted areas that afford subversive behavior
Pedophiles	Areas associated with known pedophiles
Fight spaces	Areas where public violence and fighting occur
Gang presence	Areas where gang members frequently congregate
Poor street lighting	Poorly lighted areas affording subversive behavior
Unhoused residents	Areas where unhoused (homeless) residents congregate
Los Angeles sheriff	Areas where community youth experience sheriff harassment (eg, stop and frisk)
Danger at night	Areas known to be dangerous during evening hours
Sexual harassment	Areas where female residents experienced sexual harassment
Tobacco shops	Retailers specializing in tobacco and illicit drug-use products
Off-sale alcohol outlets	Retailers selling alcohol products for consumption off-premises
Medical marijuana dispensaries	Retail locations that sell medical marijuana for medical use
Problem motels	Low-cost motels affording subversive behavior out of public view

**Figure 2:**
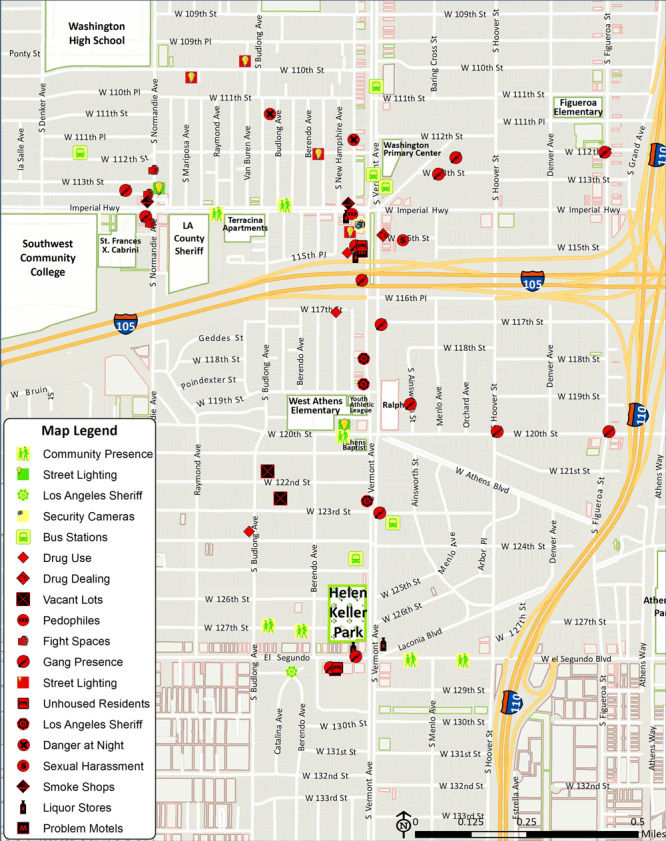
Digitized participatory geographic information systems map of community park access assets and challenges, South Los Angeles, 2015.

Residents contended that off-sale alcohol outlets compromised park accessibility and community mobility, asserting that these nuisance properties were magnets for crime and violence, a phenomenon that has been well documented in the literature (32). For example, a young resident mapped a liquor store within 130 feet of Helen Keller Park (Figure 2). Adult residents also indicated that the liquor store was a site of ongoing gang violence, including a recent murder. Furthermore, residents were concerned that patrons from a nearby problem motel synonymous with drug use and prostitution frequented the liquor store, potentially increasing exposure of park users to crime and violence (Figure 2).

Residents were also concerned about criminal activity around tobacco shops and, to a lesser extent, MMDs (although no MMDs were identified on the maps provided), with a young participant describing these properties as spaces that attract crime and violence. Although the literature at the time of our mapping project had noted property and violent crime associations with MMDs (33), crime-related research on tobacco shops was markedly absent; 1 study had documented associations between tobacco shop density and social deprivation (34), a known correlate of crime (35).

#### Step 6: Reconvene residents for map review

Twenty-eight community residents reconvened 1 month after the initial participatory mapping sessions to review digitized PGIS maps (Figure 2). This follow-up meeting was designed to 1) ensure that all resident spatial concerns about access to Helen Keller Park had been accurately mapped, and 2) discuss how to use the data. Although the Community Coalition and their community base were actively engaged in efforts to address problem motels and off-sale alcohol outlets, residents contended that tobacco shops, off-sale alcohol outlets, and MMDs comprised a trifecta of legal drug outlets warranting further investigation and community action to prevent crime and violence around these properties.

#### Step 7: Scale maps to larger geographic area

To explore validity of this novel qualitative study on a community-wide scale, our South Los Angeles REACH team, including Community Coalition staff, conducted a cross-sectional PGIS investigation of the identified legal drug outlets’ geospatial associations with crime and violence in South Los Angeles. The team accordingly acquired and mapped the following point location data (from January through December 2014) across 116 contiguous census tracts in South Los Angeles: 1) all tobacco shops, off-sale alcohol outlets, MMDs, and grocery and convenience stores that sold alcohol and tobacco, and 2) property, violent, and total crime reported by the Los Angeles police and sheriff departments. We then examined mean rates of property, violent, and total crime within 100-, 200-, 300-, 500-, and 1000-foot spatial buffers of all properties and assessed geospatial associations between our 3 legal drug outlets and crime while controlling for pertinent demographic characteristics. This quantitative analysis, detailed elsewhere (36), was the first to reveal significant clustering of property, violent, and total crime within 100- and 200-foot buffers around tobacco shops and off-sale alcohol outlets, but not MMDs, when compared with grocery and convenience stores. Geographically weighted regression analyses indicated that tobacco shops were associated with increases in property, violent, and total crime, validating community concerns gained from our mapping sessions that tobacco shops were associated with crime and violence in South Los Angeles (36). These results were a strong illustration of the process and products by which PGIS produces novel public health knowledge.

### From upstream determinants to downstream community organizing and policy interventions

Leveraging PGIS findings with PARC and Community Coalition–collected polling data indicating broad-scale resident concerns about crime and violence near tobacco shops (37), the Community Coalition mobilized residents to demand policy changes. In response, the Los Angeles County Board of Supervisors initiated a county-level motion, Assessing Nuisance Tobacco Shops (38). The Community Coalition subsequently led a series of community organizing events supported by South Los Angeles residents to further disseminate research results and advocate for policies limiting the proliferation of tobacco shops in South Los Angeles. The Los Angeles County Board of Supervisors consequently voted to draft an ordinance prohibiting tobacco shops in residential zones and proximal to sensitive land uses (39), marking a major policy win toward reducing crime and violence and other potential tobacco-related health disparities (eg, lung cancer, heart disease) in disadvantaged communities (37).

## Discussion

The case example presented here involved community residents and organizers, gang interventionists, public health and park officials, and health disparities researchers in collaboratively identifying research methods and procedures using the skills and expertise of our collective partnership. The resulting mixed-methods PGIS approach combined qualitative participatory mapping techniques involving reference maps with quantitative GIS analyses to connect resident spatial, contextual knowledge (primary data) with crime data (secondary data) to empirically confirm their experiences of place. These empirical data accordingly supported resident advocacy efforts that resulted in a major policy win that could limit proliferation of tobacco shops in Los Angeles County. We thus contend that participatory mapping is a powerful, visual mechanism of knowledge production for 1) diagnosing fundamental causes of health and disease, and 2) empowering disadvantaged communities to redress systemic inequities that manifest in health disparities through community organizing and policy change initiatives grounded in resident knowledge of place (8,9).

The empowering diagnostic approach of PGIS specifically — a unique approach to disparities research that blends qualitative participatory mapping with quantitative spatial analyses — is rooted in its ability to inform public health interventions such as community organizing and policy changes that listen to the voices of marginalized residents (13). In this context, PGIS provides a grounded, resident-centered, participatory mode for gathering information and testing hypotheses about place-based determinants of health disparities in disadvantaged communities. It is an approach that correspondingly addresses the notions that 1) place shapes health, 2) large-scale spatial epidemiology without community participation neglects resident experience of place, and 3) contemporary social science methods (eg, surveys and questionnaires) lacking a rigorous visual, place-based approach are ill equipped for making explicit connections between health and place (13,16). Furthermore, dovetailing well-established forms of participatory mapping (eg, hands-on mapping) that are perfectly matched to exploring community health assets and challenges, and identifying mechanisms of statistically observed patterns, the PGIS case example presented in this article provides a mixed-methods, exploratory sequential approach ideally suited to informing examinations of place-based determinants of health, and thus providing concrete data that confirms, refutes, or expands on ground-level reports (15,23).

The process of participatory mapping has its challenges, including significant time investments and fostering sustained resident participation. Additionally, participatory mapping generally depends on participant spatial literacy (11,12). The Community Coalition’s ongoing relationship with South Los Angeles residents and community partners as well as its ability to recruit participants with grounded spatial knowledge partially bridged these challenges. Finally, our cross-sectional examination limited our ability to infer causal relationships between nuisance properties and crime.

Taking these strengths and challenges into account, we assert that public health research and practice would benefit from integrating and adapting empowering modes of participatory mapping to diagnose place-based determinants of health. When combined with technical GIS mapping procedures in the form of PGIS, for example, this participatory approach provides communities of practice with a bifurcated methodology for innovatively and rigorously investigating the problem of health disparities. Combining these participatory modes of spatial investigation can empower community residents and public health officials to affect the root causes of health disparities through community organizing and policy change initiatives that advance health and well-being in disadvantaged communities.
